# Rapid Decreases and Performance Declines in Northeast Pacific Seamount Foundation Species Detected in an Oxygen Minimum Zone

**DOI:** 10.1111/gcb.70878

**Published:** 2026-04-27

**Authors:** Lindsay Clark, Cherisse Du Preez, Georgia Clyde, Amanda E. Bates

**Affiliations:** ^1^ Department of Biology University of Victoria Victoria British Columbia Canada; ^2^ Fisheries and Oceans Canada Institute of Ocean Sciences Sidney British Columbia Canada

**Keywords:** deep sea, foundation species, oxygen minimum zone, performance declines, photogrammetry, population decreases, seamount

## Abstract

Seamount ecosystems are increasingly exposed to rapid oceanographic change, including warming waters, declining oxygen concentrations, and the upward migration of carbonate saturation horizons. Together, these processes are compressing the depth ranges of suitable habitat for many deep‐sea organisms and altering the environmental conditions structuring benthic communities. While deep‐sea environments have historically been considered relatively stable due to low environmental variability, empirical evidence documenting how populations respond to ongoing ocean change remains scarce. Here, we use high‐resolution photogrammetric reconstructions of 12 monitoring sites (350–1111 m depth) across three Northeast Pacific seamounts to assess changes in the abundance and condition (i.e., health) of cold‐water corals and sponges. Baseline reconstructions established in 2018 were compared with repeat surveys conducted 3–5 years later. Contrary to expectations for these slow‐growing, long‐lived species, significant declines in both abundance and condition were observed. Across the 12 sites, 163 of 844 individuals were lost between surveys, with abundance declining at five sites and condition declining at nine. The most severe losses occurred at a single site on Explorer Seamount, where 51% of individuals were lost, including approximately 80% of the dominant sponge species. Sponges experienced greater declines than corals across all metrics, and the most impacted sites were not consistently located within the lowest oxygen concentrations of the expanding oxygen minimum zone. Although abundance change did not differ significantly among oxygen zones, condition scores were lower at sites with the lowest oxygen levels. These findings suggest that early impacts of ocean change may already be occurring in deep‐sea foundation species, highlighting the importance of repeat monitoring to detect rapid ecological change in environments traditionally assumed to be stable.

## Graphical abstract

Across 12 long‐term monitoring sites on three Northeast Pacific seamounts, 163 of 844 cold‐water coral and sponge individuals were lost between surveys 3–5 years apart, with abundance declining at five sites and condition (i.e., health) declining at nine. Sponges experienced greater declines than corals across all condition metrics, and condition scores were lower at sites with the lowest oxygen levels for both phyla. These results suggest that early impacts of ocean change may already be occurring in the deep‐sea, highlighting the importance of monitoring to detect rapid ecological change.

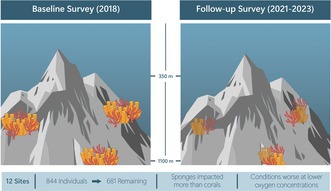

## 1 Introduction

The stability (i.e., the ability to respond to, resist, or recover from disturbance; Domínguez‐García et al. 2019), and functioning of an ecosystem are largely a result of the biodiversity it contains (Hatton et al. 2024; Hisano et al. 2018). Functional redundancy between species can provide insurance against loss (the “insurance hypothesis”; Loreau et al. 2021; Yachi and Loreau 1999), while niche complementarity can reduce the variability in species' responses to disturbance (“portfolio effects”; Schindler et al. 2015; Thibaut and Connolly 2013). Further, increased biodiversity can enhance key ecosystem processes, such production, nutrient cycling, and the efficient use of resources (Gamfeldt et al. 2015; Hooper et al. 2005; Isbell et al. 2013). The ability of biodiversity to maintain the stability and function of an ecosystem, however, is directly related to both the type and intensity of disturbance an ecosystem must withstand (White et al. 2024), as well as the sensitivity of the species involved (De Bello et al. 2021; McLean et al. 2019).

Heightened biodiversity turnover or losses within an ecosystem are therefore key signals to shifting stability and functioning (Hautier et al. 2015; Spaak et al. 2017) and often are the result of species' range shifts in response to environmental change (Brodie et al. 2025; Pecl et al. 2017). Contractions along the unfavourable edge of a range (trailing edge), where biodiversity losses are most likely to occur, can conceptually be broken into three stages (Bates et al. 2014). Initial declines in organismal performance, such as negative changes in growth, condition (i.e., health), or reproductive success, are expected with shifting environmental conditions. As condition changes persist or worsen, population effects can be expected as recruitment rates, emigration, and survivorship declines (Bates et al. 2014; Poloczanska et al. 2016; Wernberg et al. 2010). If successive declines in growth, health, and reproductive success continue over time, recruitment rates are unable to compensate for losses, and population decreases can ultimately lead to local extinction. Evidence for performance declines, population decreases, and local extinctions in the marine environment have thus far been largely attributed to heat stress (e.g., Edgar et al. 2023; Wiens 2016) and are generally sourced from coastal environments (e.g., Smale and Wernberg 2013).

Knowledge of biodiversity changes due to non‐temperature oceanographic change and from remote habitats is limited. The deep sea, historically characterized by remarkably stable environmental conditions, is dominated by highly adapted species with narrow niche requirements (Danovaro et al. 2017; Paulus 2021). Further, food is often limited within these ecosystems and combined with the effects of extreme pressure and frigid temperatures, the pace of life for deep sea organisms is often slow, with decadal generation times (e.g., COSEWIC 2007; Parker et al. 1997) and lifespans lasting centuries in some species (e.g., Leys and Lauzon 1998; Love et al. 2007; Risk et al. 2002). Together, these life history traits make deep‐sea organisms particularly susceptible to disturbances and environmental change (Smith et al. 2008). Recent studies have increasingly shown that rapid environmental change associated with climate change is already impacting, or expected to impact, deep‐sea ecosystems, threatening biodiversity, ecosystem stability, and ecosystem functioning (Morato et al. 2020; Sweetman et al. 2017), with recent evidence documenting significant declines in deep‐sea foundation species (Rakka et al. 2025). In the Northeast Pacific, where oceanographic time‐series data collected along a fixed transect that has been resampled for decades (Line P; Freeland 2007), oxygen content declines are exacerbating a naturally present oxygen minimum zone (OMZ; 3.0 ± 0.7 m/year; Ross et al. 2020). These increasingly hypoxic conditions threaten biodiversity on the region's seamounts, either through direct exposure to deoxygenated waters (many seamounts transect the OMZ, which stretches from ~480 to 1700 m depth; Ross et al. 2020), or from secondary impacts, such as the alteration of export productivity (i.e., changes in the flux of organic matter) from surface waters (Du Preez and Norgard 2022; Ross et al. 2020).

Changes in dissolved oxygen are of particular concern for sessile, habitat‐forming foundation species such as cold‐water corals (Cnidaria) and sponges (Porifera) (Leys et al. 2004; Thresher et al. 2011). As the protection, conservation, and restoration of these deep‐sea foundation species are among the goals of the region's Marine Protected Areas (MPAs; Du Preez et al. 2024), monitoring for performance declines (i.e., negative changes in growth, health condition, or reproductive success: Bates et al. 2014), population decreases, and, ultimately, range contractions is necessary. However, monitoring of remote deep‐sea habitats can be both challenging and costly, ultimately limiting the knowledge of climate‐related biodiversity changes needed to inform effective MPA management (Juniper et al. 2019). Additionally, while abundance or presence‐absence measures are effective for detecting local extinctions and population decreases, performance declines are often missed by these metrics (Bates et al. 2014).

Sampling in remote environments has expanded due to recent technological advancements. Here we use Structure‐from‐Motion (SfM) photogrammetry to efficiently monitor deep‐sea habitats. SfM photogrammetry uses superimposed, overlapping images to create high resolution reconstructions of surfaces (Westoby et al. 2012), and has gained popularity for use in both shallow‐ and deep‐water research to characterize habitat metrics (e.g., structural complexity, slope, rugosity; Bruce 2021; Burns et al. 2019; Prado et al. 2020), investigate and monitor community structure (Burns et al. 2015; Heres et al. 2024; Van Audenhaege et al. 2024), and detail growth and climate‐related changes (Bennecke et al. 2016; Fukunaga et al. 2022; Olinger et al. 2019). While these studies demonstrate the potential of photogrammetric approaches for monitoring, repeated sampling of discrete deep‐sea coral and sponge habitats across multiple sites remains rare, limiting the ability to detect population and condition changes over time and establish critical ecological baselines and effective indicators to inform conservation strategies.

Here, we calculate abundance and condition metrics for cold‐water corals and sponges in long‐term monitoring sites on three seamounts between two time points, 3–5 years apart, while establishing ecological baselines for continued monitoring. While climate‐related abundance and condition declines in cold‐water corals and sponges can occur, declines may lag and remain undetected for decades because corals and sponges live on the slow continuum at cold temperatures (Du Preez et al. 2024; Kenchington 2010). As such, we expected very little detectable change in the abundance and condition of species over the study time frame (≤ 5 years). Even so, if signs of population declines are detected we predict that these will occur within the nearly anoxic core of the OMZ (~< 0.5 mL/L O_2_, 800–1200 m; Ross et al. 2020), where oxygen concentrations are approaching the limit for sustaining deep‐sea megafauna (Gooday et al. 2010; Wishner et al. 1990). We further expect declines, if observed, for the most sensitive and exposed species. Sponges, which can tolerate hypoxic conditions but at the cost of feeding (Leys and Kahn 2018; Riisgård 2024), may be particularly sensitive to prolonged exposure to low oxygen concentrations.

## 2 Methods

### 2.1 Long‐Term Monitoring Sites

**FIGURE 1:**
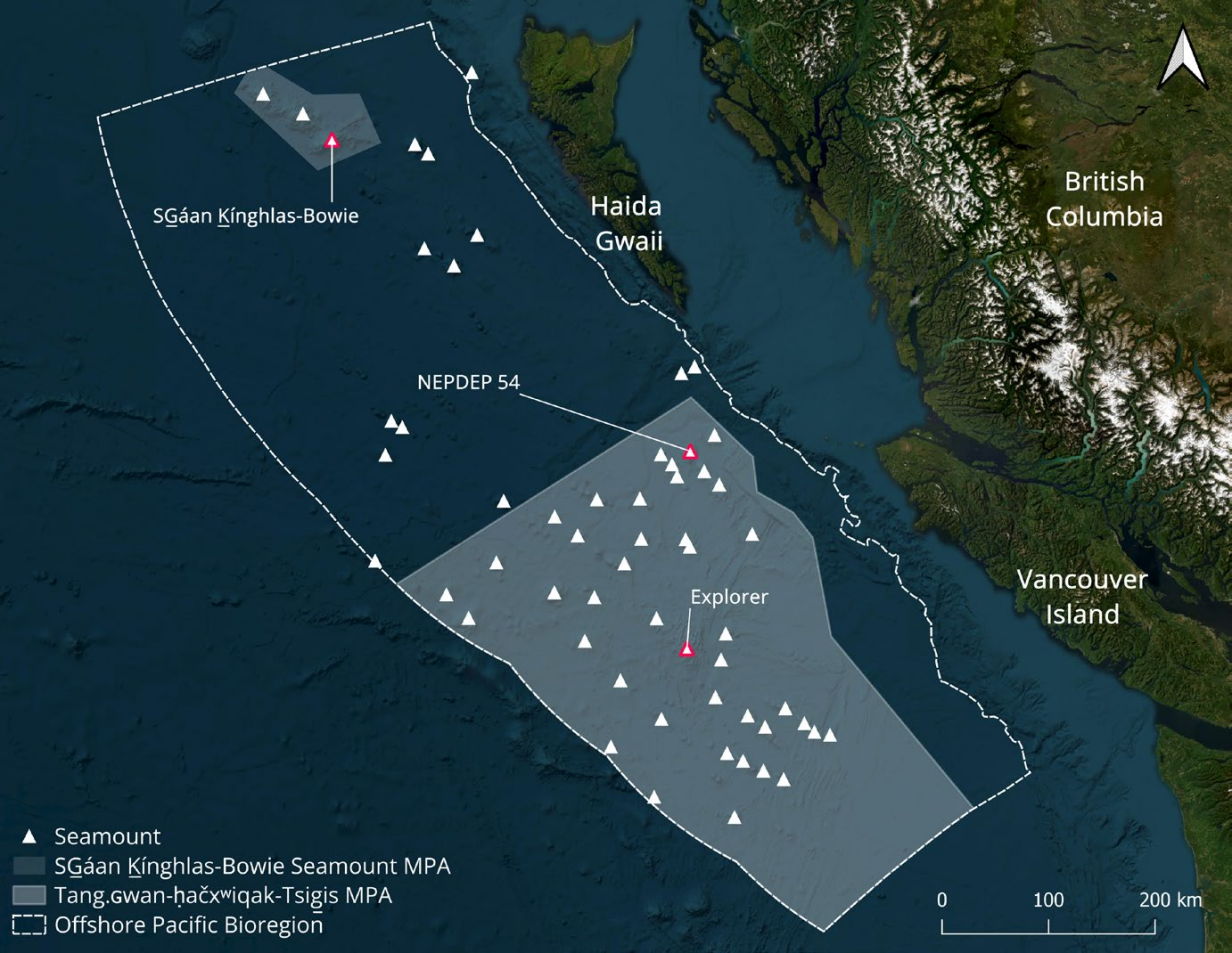
Northeast Pacific seamounts and deep‐sea Marine Protected Areas (MPAs) within the offshore area of the Canadian Exclusive Economic Zone (Offshore Pacific Bioregion) and territorial waters of the Haida, Nuu‐chah‐nulth, Quatsino, and Pacheedaht Nations. The three study seamounts, SG̲áan K̲ínghlas‐Bowie, NEPDEP 54, and Explorer, are highlighted in red.

The 2018 Northeast Pacific Seamount Expedition used the E/V *Nautilus* and the remotely operated vehicle (ROV) *Hercules* to set up and survey 12 long‐term monitoring sites (~10 m by 10 m) on three Northeast Pacific seamounts (Gartner et al. 2022; Table 1). Seven sites are located on two seamounts within the Tang.ɢwan‐ḥačxʷiqak‐Tsig̱is (TḥT) MPA (NEPDEP 54, formerly Dellwood: 5 sites, Explorer: 2 sites), and five sites are on SG̲áan K̲ínghlas‐Bowie (SK̲‐B) seamount within the SK̲‐B Seamount MPA (Du Preez and Norgard 2022; Figure 1). These monitoring sites were placed at depths exposed to the OMZ (~480–1700 m) and in areas with high abundances of corals and sponges (Gartner et al. 2022). All 12 sites were initially imaged in 2018 and then revisited for the first time between 3 and 5 years post‐establishment, with all repeat surveys conducted aboard the CCGS *John P. Tully* (Table 1). In 2021, sites on NEPDEP 54 were resurveyed during the 2021 Northeast Pacific Seamount Expedition with the Fisheries and Oceans Canada (DFO) drop camera system BOOTS (Bathyal Ocean Observation and Televideo System). Sites on SK̲‐B were resurveyed in 2022 using ROV *Odysseus*, and, in 2023, the sites on Explorer seamount were resurveyed with ROV *ROPOS*.

**TABLE 1:** Long‐term monitoring sites on NEPDEP 54, SG̲áan K̲ínghlas‐Bowie (SK̲‐B), and Explorer seamounts, re‐surveyed for foundation species abundance and condition between 2021 and 2023. All sites were established in 2018 as a part of the 2018 Northeast Pacific Seamount Expedition. Mean oxygen concentrations (mL/L) were determined using measurements collected by the ROV or drop camera during site surveys.

Seamount	Survey years	Long‐term monitoring site	Latitude (°)	Longitude (°)	Depth (m)	Mean oxygen conc. (mL/L)	
						T_0_	T_1_
NEPDEP 54	2018, 2021	N54‐01	50.722	−130.921	833	0.15	0.27
		N54‐02	50.757	−130.888	625	0.30	—
		N54‐03	50.7577	−130.886	640	0.23	—
		N54‐04	50.757	−130.887	633	0.24	—
		N54‐06	50.757	−130.889	607	0.29	—
SG̲áan K̲ínghlas‐Bowie	2018, 2022	SK̲‐B‐06	53.322	−135.536	1111	0.15	0.39
		SK̲‐B‐07	53.322	−135.562	644	0.32	0.39
		SK̲‐B‐08	53.321	−135.545	828	0.14	0.34
		SK̲‐B‐11	53.281	−135.765	467	0.87	0.95
		SK̲‐B‐12	53.28	−135.763	350	1.43	1.22
Explorer	2018, 2023	EX‐01	49.058	−130.942	799	0.13	0.25
		EX‐02	49.058	−130.94	868	0.13	0.25

### 2.2 Image Acquisition and 3D Reconstruction

For all monitoring sites, video imagery of the seafloor was collected using forward‐facing HD cameras (*Hercules* and *ROPOS*: Insite Pacific Zeus Plus, BOOTS and *Odysseus*: Insite Pacific Mini Zeus) with 10 cm scaling laser projections, and positional data was collected using acoustic navigation sensors (Canadian Scientific Submersible Facility 2026; Gale et al. 2017; Ocean Exploration Trust 2014; Pelagic Research Services 2026). Surveys in 2018, 2022, and 2023 followed a “mow‐the‐lawn” protocol to collect overlapping imagery of each site (Gartner et al. 2022), while the 2021 survey utilized an opportunistic single transect “fly‐by” method. All surveys maintained a vehicle speed over ground between ~0.1 and 0.3 knots, while the ROV surveys maintained a relatively constant heading (variation within ~10 degrees) and altitude (or depth, if tall organisms were present in the site; average altitude: 3.5 m) unique to each site (established in 2018 and repeated in subsequent years). Heading and altitude during the 2021 drop camera survey were variable due to equipment limitations. All raw video imagery data is stored and available through Ocean Networks Canada's SeaTube Pro (Ocean Networks Canada 2026).

For the reconstructions, still images were extracted from video imagery at a rate of 1 every 2 s using ImageCaptureTool, a Python script developed by DFO's Marine Spatial Ecology and Analysis (MSEA) group for use in image annotation projects (Martin and Nephin 2019). Colour corrections (white balance with adjustment to contrast and brightness, as needed) were applied to still images using GNU Image Manipulation Program (GIMP; The GIMP Development Team 2023) and the Batch Image Manipulation Plugin (BIMP; Francesconi 2021) for batch‐processing. Images were manually reviewed to remove those with motion blur, ROV equipment, or obstructions (e.g., fish) in the frame. For all reconstructions excluding those derived from 2018 imagery of sites on NEPDEP 54, ROV position [i.e., latitude, longitude (WGS84/World Mercator projection), and depth (m)] at time of image capture was appended to the EXIF metadata of each image to optimize the georeferencing and processing time of reconstructions. To do so, a purpose‐built workflow was created utilizing the programming language R (R Core Team 2024) and RStudio (Posit team 2023), EXIFTOOL (Harvey 2022), and Geosetter (Schmidt 2020).

Still images were imported into Pix4Dmapper (Pix4D 2022), a software that uses SfM methods to create reconstructions based on image contents, camera specification (e.g., focal length, sensor size), and user defined settings. Similar to methods used by Gerdes et al. (2019), all photogrammetric reconstructions were created using the software's preset “3D Map” setting, the WGS84/World Mercator coordinate system to match ROV navigational data, and the internal units were set to meters. Additional settings, such as the maximum number of image pairs, were optimized for each individual reconstruction to produce the highest quality reconstruction possible. Scaling laser projections within the still images were used to apply scaling constraints to the reconstructions and tie points were created using vehicle navigation data attributed to easily identifiable seafloor features (e.g., site marker) at the corners of each reconstruction to assist with georeferencing and decrease processing time. Vehicle navigation data was obtained for each tie point when the feature in question was located centrally along the bottom edge of the image frame (closest position to vehicle sensors) within the raw video imagery. Scaling constraints were not applied to reconstructions using 2021 drop camera video footage, as the imagery quality was too low (e.g., blurred, grainy) to determine suitable features for applying constraints. Additionally, navigation data was not used to georeference reconstructions for sites on NEPDEP 54 surveyed in 2018.

While each reconstruction generated multiple outputs, only the two‐dimensional mosaics were used herein. The two‐dimensional mosaics were selected for organism annotation because of the increased support of this datatype across GIS platforms, including the open‐source QGIS (QGIS 2023), the decreased processing requirements, and the ease of comparing annotations between T_0_ and T_1_. Further, for each reconstruction, two‐dimensional mosaics were manually edited in Pix4D, using planimetric visual corrections, to increase visibility of seafloor features and organisms, thereby increasing the accuracy of organism identification and improving alignment between T_0_ and T_1_ mosaics.

### 2.3 Oxygen Concentration Zones

Raw dissolved oxygen data were recorded for each long‐term monitoring site surveyed during T_0_ (2018) and T_1_ (site specific) using an Aanderaa 3830 oxygen optode (ROV *Hercules*; Ocean Exploration Trust 2014) or a Sea‐Bird Scientific SBE43 dissolved oxygen sensor (ROV *Odysseus*, ROV *ROPOS*, & BOOTS; Gale et al. 2017; L. Girard, personal communication, March 10, 2026). Unfortunately, oxygen data for four of the five NEPDEP 54 sites re‐surveyed in 2021 cannot be used due to calibration issues.

We used the video imagery time stamps from the 3D reconstructions and calculated an average oxygen concentration for each site at T_0_ and T_1_ (where possible). Using the T_0_ mean, sites were classified into one of three concentration zones used in Ross et al. (2020): [O_2_] > 1 mL/L, 1 > [O_2_] > 0.5 mL/L, and [O_2_] < 0.5 mL/L. Raw oxygen profiles at T_0_ and T_1_ were not directly compared due to uncertainty surrounding precise absolute measurements between different instruments. However, T_0_ and T_1_ [O_2_] averages were compared to determine if sites changed their zone over time.

### 2.4 Organism Annotations

The two‐dimensional mosaics were imported into the open‐source GIS platform QGIS (QGIS 2023) and, using the georeferencing plugin, mosaics from 2021 to 2023 (T_1_) were aligned to the 2018 mosaics (T_0_) using 1st‐order polynomial transformations and the root mean squared error (RMSE) for georeferencing (cm) and area of overlap (m^2^) was calculated for all sites.

All coral and sponge individuals > 5 cm (estimated using scaling laser projections) were identified to the lowest operational taxonomic unit (OTU) possible, with confidence, based on a regional photographic guide (Du Preez et al. 2020) and annotated. For T_1_ mosaics, only individuals present in areas overlapping the T_0_ mosaics were annotated. All annotations included information on the organism's identity (phylum, OTU) and location (latitude, longitude, depth), as well as the site, seamount, and timepoint (T_0_ or T_1_). Additionally, organisms within the T_0_ mosaics were assessed a condition score of zero, as the T_0_ mosaics serve as a baseline for all future monitoring of these sites.

**FIGURE 2:**
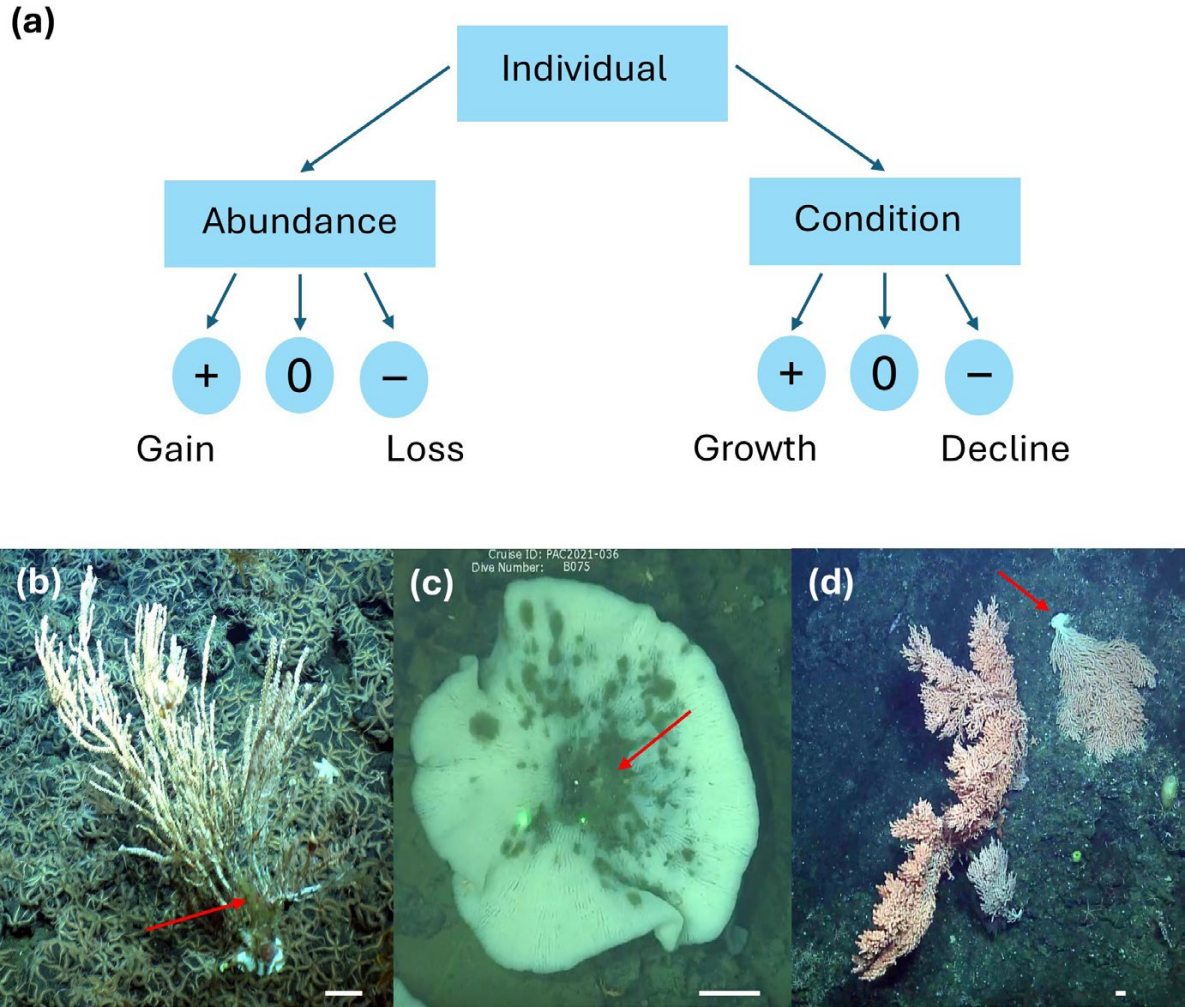
Illustration of how individuals were assessed for abundance and condition changes over time (a), with examples of negative condition metrics, including biofouling on an *Isidella tentaculum* in monitoring site SK̲‐B‐07 in 2018 (b), tissue loss on a *Tretodictyum* n. sp. glass sponge in site N54‐01 in 2021 (c), and horizontal orientation change of a *Primnoa pacifica* coral in site SK̲‐B‐12 in 2018 (d). 10 cm white scale bars are in the bottom right corner of (b–d), while red arrows indicate the condition metric of interest.

For the T_1_ condition scores, all corals and sponges were qualitatively assessed on four condition metrics: growth (including regrowth), tissue loss, biofouling (i.e., growth of other organisms on the body), and vertical orientation change (e.g., slanted or toppled; Figure 2). Growth was the only metric representing positive condition change, while tissue loss, bio‐fouling, and vertical orientation change represent negative condition changes. While uncertainties exist as to the extent of vertical orientation changes' impact on coral and sponge health, particularly in high flow environments such as seamounts, deviations in this metric were included as a negative condition change as current research suggests decreased elevation from the seafloor increases the influence of the benthic boundary layer on individuals and thus reducing food delivery and availability (Buhl‐Mortensen et al. 2010; Sebens et al. 2017) and increasing predation potential (i.e., through easier access for benthic predators).

Assessments for each metric were made through comparisons of the two‐dimensional photomosaics and raw imagery used for the reconstructions. For all metrics except vertical orientation change, metrics were scored from 0 to 4 based on increased change between T_0_ and T_1_ (0: 0%, 1: 1%–25%, 2: 26%–50%, 3: 51%–75%, 4: 76%–100%). Scores for vertical orientation change ranged from 0 to 2, with 0 indicating an individual that experienced no change between T_0_ and T_1_, 1 indicating a shift between approximately 1°–45° from its T_0_ orientation, and 2 representing an individual that had shifted ≥ ~46°.

### 2.5 Data Analysis and Statistics

Organism annotations were imported in R (R Core Team 2024) for analysis. To account for the sampling bias toward negative metrics (i.e., three measures of negative change versus one for positive change), as well as the disparity between rates of growth, recovery, and decline in deep‐sea species (e.g., Girard et al. 2018; Girard and Fisher 2018; Leys and Lauzon 1998), initial metric scores were scaled between zero and one and frequency weighted by score occurrence, similar to methods used for prioritization analyses of trait‐based climate change vulnerability frameworks (Potter et al. 2017; Richards et al. 2022). For example, the scaling and weighting steps used for metrics scored from 0 to 4 (i.e., growth, tissue loss, and biofouling) were applied as follows: initial score of 0 = 0, initial score of 1 = *n*_1_/*n*_total_, initial score of 2 = (*n*_1_ + *n*_2_)/*n*_total_, initial score of 3 = (*n*_1_ + *n*_2_ + *n*_3_)/*n*_total_, initial score of 4 = (*n*_1_ + *n*_2_ + *n*_3_ + *n*_4_)/*n*_total_ = 1, where n is the number of individuals with an initial score (1–4), and *n*_total_ is the total number of individuals observed with a change in condition between T_0_ and T_1_. The above equations were applied to each metric independently and only used the initial scores present for each metric within the dataset (e.g., growth initially scored between 0 and 4, but only scores of 0–3 were recorded).

Weighting was applied to each metric individually; however, weighting alone does not preserve the relative severity of scores within a metric. For example, if the maximum biofouling observed across all individuals was 1%–25% (initial score = 1), a weighted score based on this value could exceed the weighted score of an individual with 51%–75% tissue loss (initial score = 3) if biofouling carried a higher weight. To address this, a severity scaling step was applied after weighting, ensuring that scores remained proportional to their original severity ranking within each metric. Each weighted score was therefore scaled as follows: severity‐scaled score = weighted score × (S/S_max_), where S is the initial score assigned to the individual for that metric, and S_max_ is the maximum possible initial score for that metric (4 for growth, tissue loss, and biofouling; 2 for vertical orientation change). Severity‐scaled scores for negative metrics were then multiplied by −1, and scores were summed across all metrics to produce an overall condition score ranging from −1 to 1. Weighted and scaled metric scores are reported in Table S1.

To test for changes in abundance and condition, abundances at T_0_ and T_1_ were calculated for each species/taxon (OTU), while baseline condition scores at T_0_ (0) were compared to the average condition score for each OTU at T_1_. Paired Wilcoxon signed‐rank tests (exactRankTests package; Hothorn and Hornik 2022) using the “greater than” alternative null hypothesis and Bonferroni *p*‐value adjustment method to correct for multiple comparisons were used to determine if the abundance and condition of corals and sponges within the long‐term monitoring sites were significantly greater at T_0_ compared to T_1_.

Abundance and condition changes over time were also compared between sites, phyla, oxygen concentration zones, and seamounts. For abundance, the % change in abundance (Δ abundance) per OTU was calculated for each factor (e.g., site, phylum) as: A_1_—A_0_/A_0_. where A is the number of individuals of an OTU present within a factor at T_0_ or T_1_. For example, an OTU that loses 10 of the 100 individuals present at T_0_ within a site would have a Δ abundance of −10% for that site, while, at the phylum‐level, if only 10 individuals of the OTU are lost from the 200 observed across all sites, the Δ abundance would be −5%. The condition score of all individuals within a factor (i.e., condition scores are not summed or averaged across OTUs) were used to compare changes in condition across sites, phyla, oxygen concentrations, and seamounts.

Kruskal Wallis tests (rstatix package; Kassambara 2023) or Wilcoxon signed‐rank tests, where appropriate, were used to determine if significant differences in Δ abundance and condition scores existed between factors (e.g., sites, phyla) and a significance level (α) of 0.05 was used for all tests. In the event of a significant result from the Kruskal Wallis tests, a *post hoc* Dunn's test was performed (rstatix package) to check pairwise comparisons with adjusted significance levels to reduce false positive errors. Finally, Wilcoxon signed‐rank tests were used to examine differences in Δ abundance and condition scores between phyla with respect to oxygen concentration zones.

## 3 Results

### 3.1 Quality of 3D Reconstructions

**FIGURE 3:**
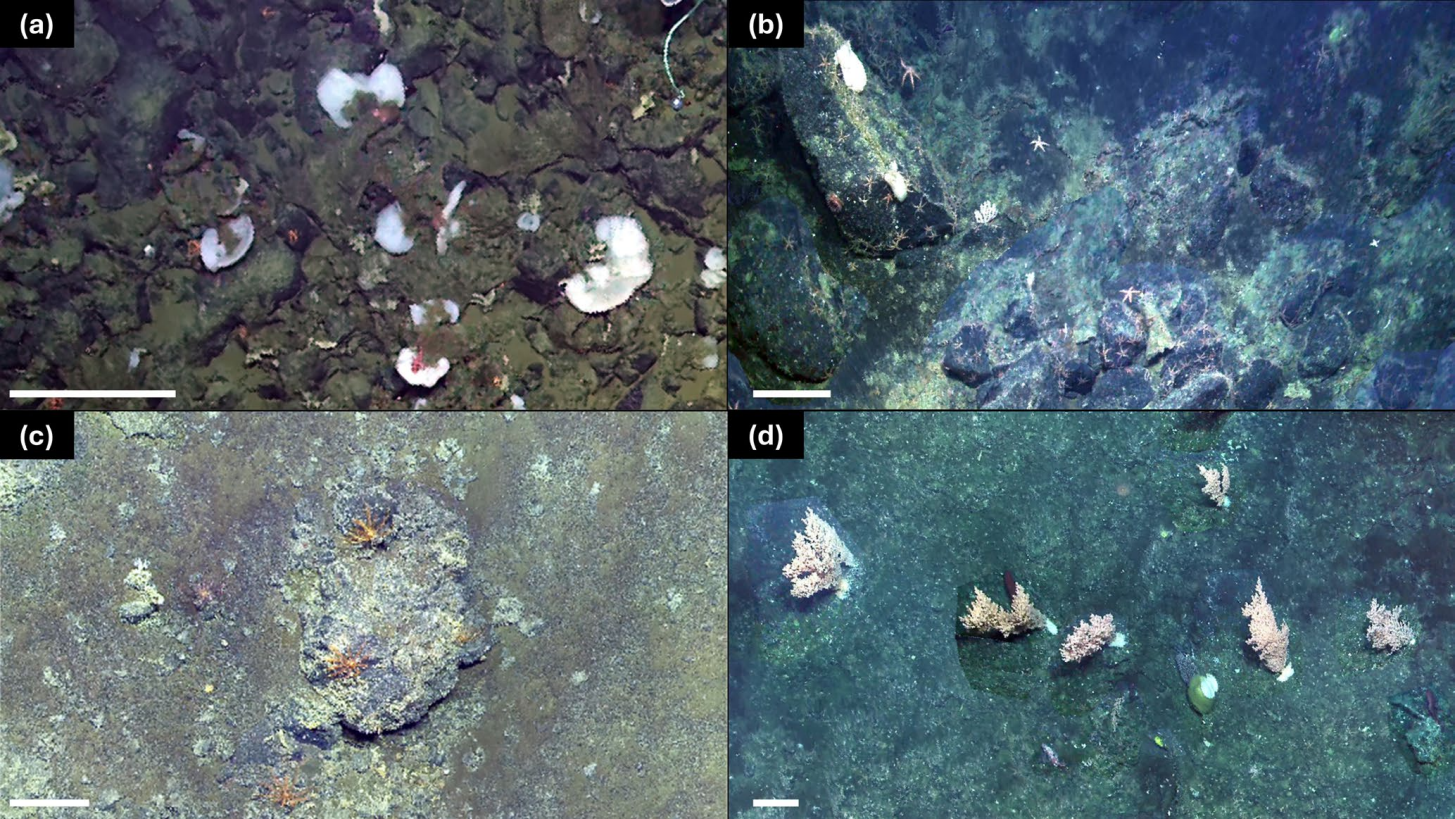
Example images of two‐dimensional mosaics derived from reconstructions of long‐term monitoring sites on Northeast Pacific seamounts, utilizing 2018 ROV video footage: N54‐01 (a), N54‐03 (b), SK̲B‐06 (c), SK̲B‐11 (d). Scale bars represent 50 cm.

We created a total of 24 reconstructions using imagery from 19 ROV (*Hercules*, *Odysseus*, and *ROPOS*) and five drop camera (BOOTS) dives (Figure 3; Table S2). ROV reconstructions (i.e., derived from surveys in 2018, 2022, and 2023) enhanced detectability along the seafloor with higher resolution (0.38 ± 0.12 cm/pixel) compared to drop camera reconstructions (0.52 ± 0.30 cm/pixel). The initial input camera parameters compared to the optimized internal camera parameters differed more for reconstructions created with ROV imagery (5.0% ± 3.8%) compared to BOOTS reconstructions (1.6% ± 0.89%). For all reconstructions with scaling constraints applied, computed error length was low (0.040 ± 0.080 m), while reconstructions using ROV positional data had an absolute geolocation RMSE of 0.19–4.2 m in the x‐direction, 0.13–5.2 m in the y‐direction, and 0.086–3.6 m in the z‐direction. The area covered by each reconstruction, determined using 2018 imagery and setting a baseline for all future surveys, varied between ~120 and 370 m^2^ (Table S3). In subsequent years, the lowest site coverage occurred using the opportunistic “fly‐by” drop camera method in 2021 (2021: 7.0%–43% of 2018 site area, 2022 and 2023: 75%–100% of 2018 site area). When aligning T_0_ and T_1_ mosaics, the georeferencing RMSE ranged from 0.034 to 0.97 m with an average of 0.36 ± 0.35 m.

### 3.2 Mean Oxygen Concentration

In 2018 (T_0_) when the long‐term monitoring sites were established, the ROV oxygen sensors recorded oxygen concentrations ranging between 0.13 (EX‐01, EX‐02) to 1.43 mL/L (SK̲‐B‐12; Table 1). At T_1_, oxygen concentration values at eight of the 12 sites ranged from 0.25 (EX‐01, EX‐02) to 1.22 (SK̲‐B‐12). All sites remained within the oxygen concentration zones derived from Ross et al. (2020) ([O_2_] > 1 mL/L, 1 > [O_2_] > 0.5 mL/L, [O_2_] < 0.5 mL/L) assigned based on T_0_ measurements.

### 3.3 Abundance Changes

**FIGURE 4:**
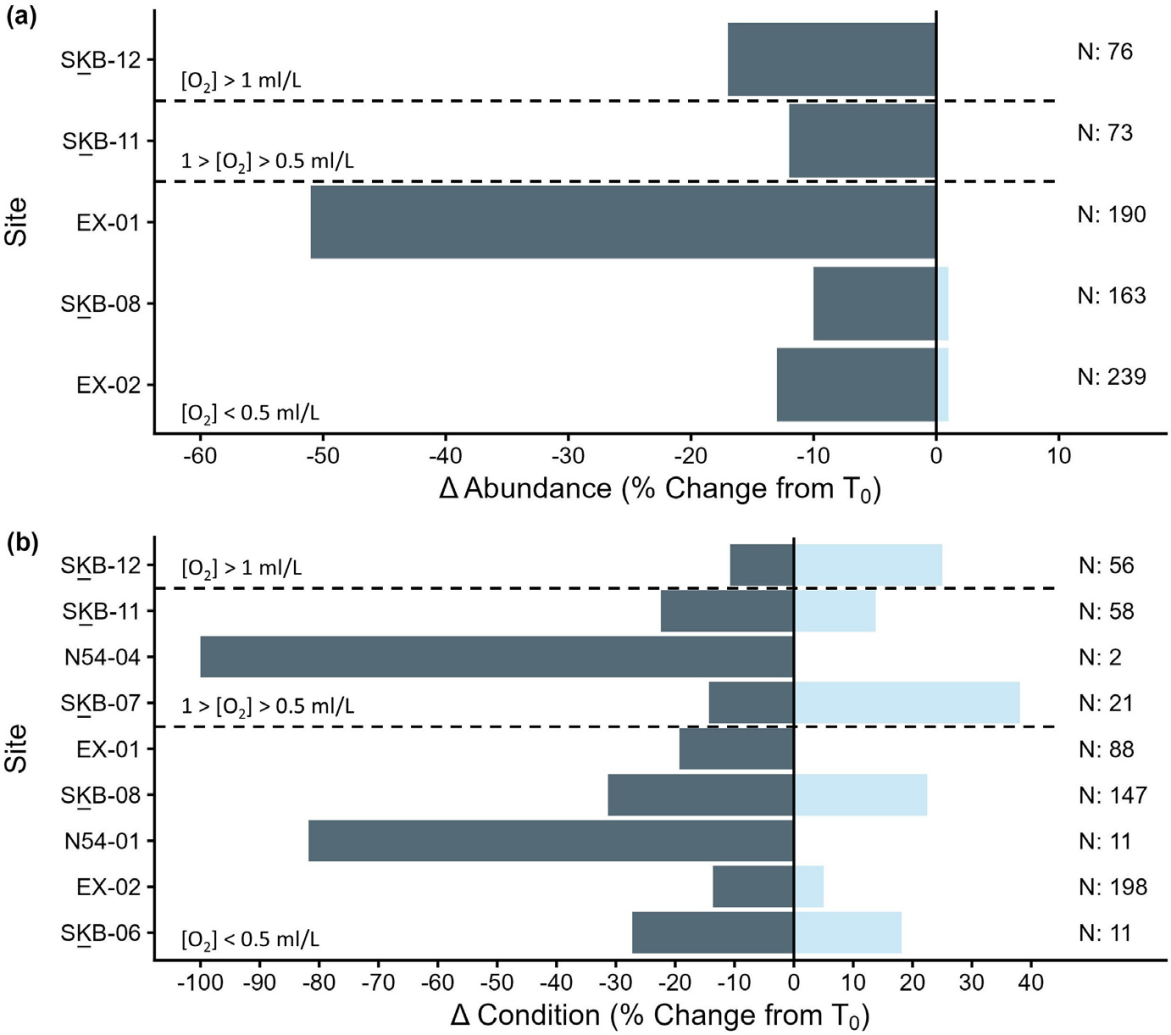
The change (%) in abundance (a) and condition (b) of cold‐water corals and sponge in long‐term monitoring sites on three Northeast Pacific seamounts over a 3–5 year period. Dark bars indicate negative change, while light bars indicate positive change. N represents the number of individuals observed at T_0_ (a) or the number of individuals with a condition change at T_1_ (b). Only sites where changes in abundance or condition between T_0_ and T_1_ occurred are included, with sites arranged in order of descending depth. Dashed lines separate sites into oxygen concentration zones based on Ross et al. (2020): [O_2_] > 1 mL/L, 1 > [O_2_] > 0.5 mL/L, [O_2_] < 0.5 mL/L.

**FIGURE 5:**
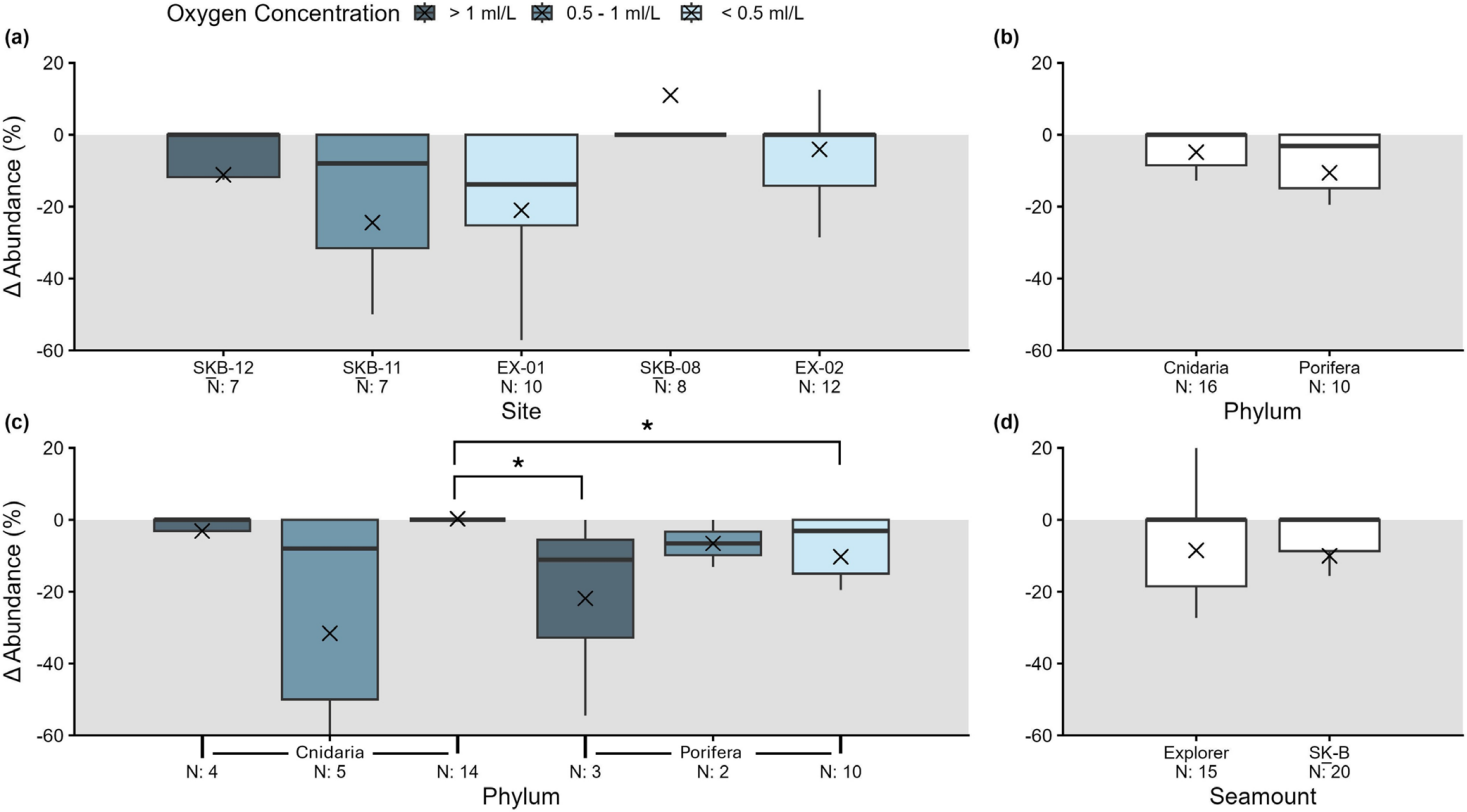
Abundance changes between T_0_ (2018) and T_1_ (site dependent) observed in long‐term monitoring sites (a) for cold‐water corals (Cnidaria) and sponges (Porifera) (b, c) situated on two Northeast Pacific seamounts: Explorer, and SG̲áan K̲ínghlas‐Bowie (SK̲‐B) (d). Sites are arranged in descending depth (left to right), N is the number of operational taxonomic units (OTUs) assessed per factor (i.e., site, phylum, seamount), and the colour scale represents oxygen concentrations as defined by concentration thresholds for the region's Oxygen Minimum Zone in Ross et al. (2020). Asterisks (*) indicate significant differences as determined by statistical analyses (*: *p* ≤ 0.05, **: *p* ≤ 0.01, ***: *p* ≤ 0.001).

Abundance declined at five of the 12 long‐term monitoring sites investigated, with 163 of 844 individuals lost between T_0_ to T_1_, averaging at ~20% of individuals per site (Figure 4a, Table S4). Across all sites (*n* = 12), abundances per OTU were determined to be significantly greater at T_0_ than T_1_ via a paired Wilcoxon signed‐rank test (*p*‐value = 0.002). Despite this, no significant differences in Δ abundance (% change per OTU) were detected between sites (Kruskal–Wallis *p*‐value: 0.160; Figure 5), phyla (Wilcoxon *p*‐value: 0.217), oxygen concentration zones (Kruskal–Wallis *p*‐value: 0.184), or seamounts (Kruskal–Wallis *p*‐value: 0.092).

**FIGURE 6:**
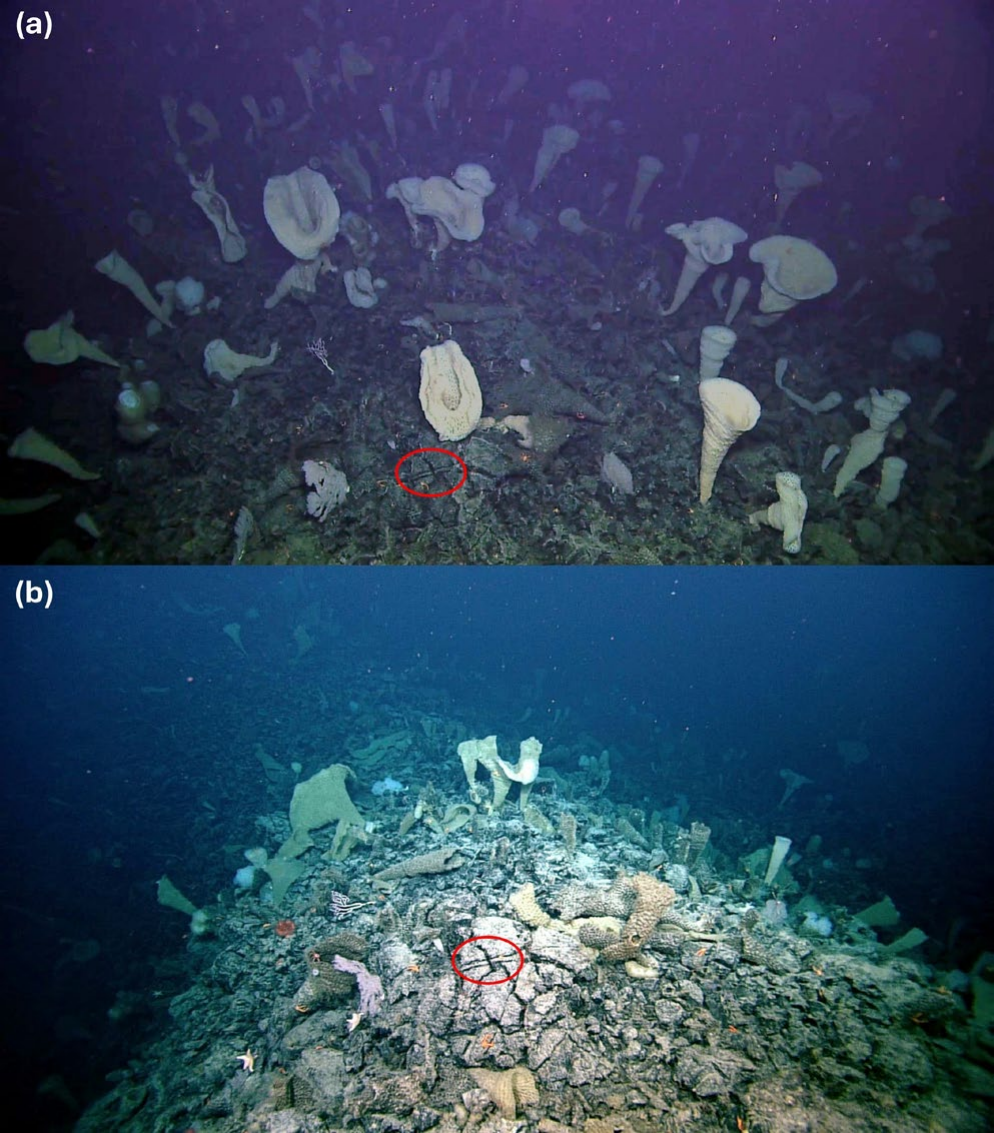
Example of severe cold‐water coral and sponge abundance losses and condition declines between 2018 (a) and 2023 (b) at site EX‐01 on Explorer seamount. 97 individuals (51%) were lost from EX‐01 over a five‐year period, with ~20% of remaining individuals experiencing negative condition changes in that time. The dominant species, *Pinulasma* n. sp. (Henry's Bugle Sponge), suffered significant losses, with a ~80% decline in abundance and 20% of remaining individuals suffering negative condition changes. The red circle marks the same striated bedrock in the two images.

For the five sites with abundance changes detected, recruitment was observed at four sites (SK̲‐B‐08: +1 individual, EX‐01: +1 individual, EX‐02: +4 individuals, SK̲‐B‐11: +6 individuals), while abundance decreases occurred at all five (Figure 4a). Losses ranged from ~10% of individuals over 4 years at SK̲‐B‐08 (15 of 73 individuals present at T_0_) to 51% of individuals between 2018 and 2023 at EX‐01 (98 of 190 individuals present at T_0_; Figure 6). At the site‐level, Δ abundance (% change per OTU) ranged between 11 ± 13% (mean ± 1 SE; SK̲‐B‐08) and −24% ± 14% (SK̲‐B‐11; Figure 5a).

Most of the individuals lost at EX‐01 were sponges (T_0_: 172, T_1_: 79), with the most abundant species, *Pinulasma* n. sp., particularly impacted (T_0_: 100, T_1_: 21). For all sites, however, abundance decreases were similar across both phyla, with 27% (143 of 527) of sponges and 10% (32 of 317) of corals lost between T_0_ and T_1_, impacting 10 of 26 OTUs (5 OTUs/phyla). Δ abundance (% change per OTU) was −4.9% ± 3.5% for Cnidarians and −11% ± 5.1% for Poriferan OTUs (Figure 5b). Sponge OTUs were impacted most at sites with the [O2] > 1 mL/L zone (−22% ± 17%), while corals suffered increased abundance declines per OTU at sites with mean oxygen concentrations between 1 and 0.5 mL/L (−32% ± 20%; Figure 5c). Significant differences in Δ abundance (% change per OTU) were found between phyla for sites [O_2_] < 0.5 mL/L (*p*‐value: 0.023) and between corals within [O_2_] < 0.5 mL/L sites and sponges within [O_2_] > 1 mL/L sites (*p*‐value: 0.040).

When considering oxygen concentration zones alone, changes in abundance were observed for all three zones, with the greatest individual losses at sites within the [O_2_] < 0.5 mL/L group, averaging at 24% of individuals lost per site (SK̲B‐08: 10%, EX‐01: 51%, EX‐02: 12%). Δ abundance (% change per OTU), however, was greatest at the lone site within the 1 > [O_2_] > 0.5 mL/L group (SK̲B‐11: −24% ± 14%). Conversely, abundance changes were only observed for long‐term monitoring sites on two of three seamounts considered herein. For sites on SK̲‐B seamount, OTUs experienced an average of −10% ± 5.4% change from T_0_, while long‐term monitoring sites on Explorer seamount had −8.5% ± 4.6% change in abundance per OTU (Δ abundance), driven by the heavy losses at EX‐01. Again, no significance was detected for Δ abundance for either oxygen concentration zones or seamounts.

### 3.4 Changes in Condition

**FIGURE 7:**
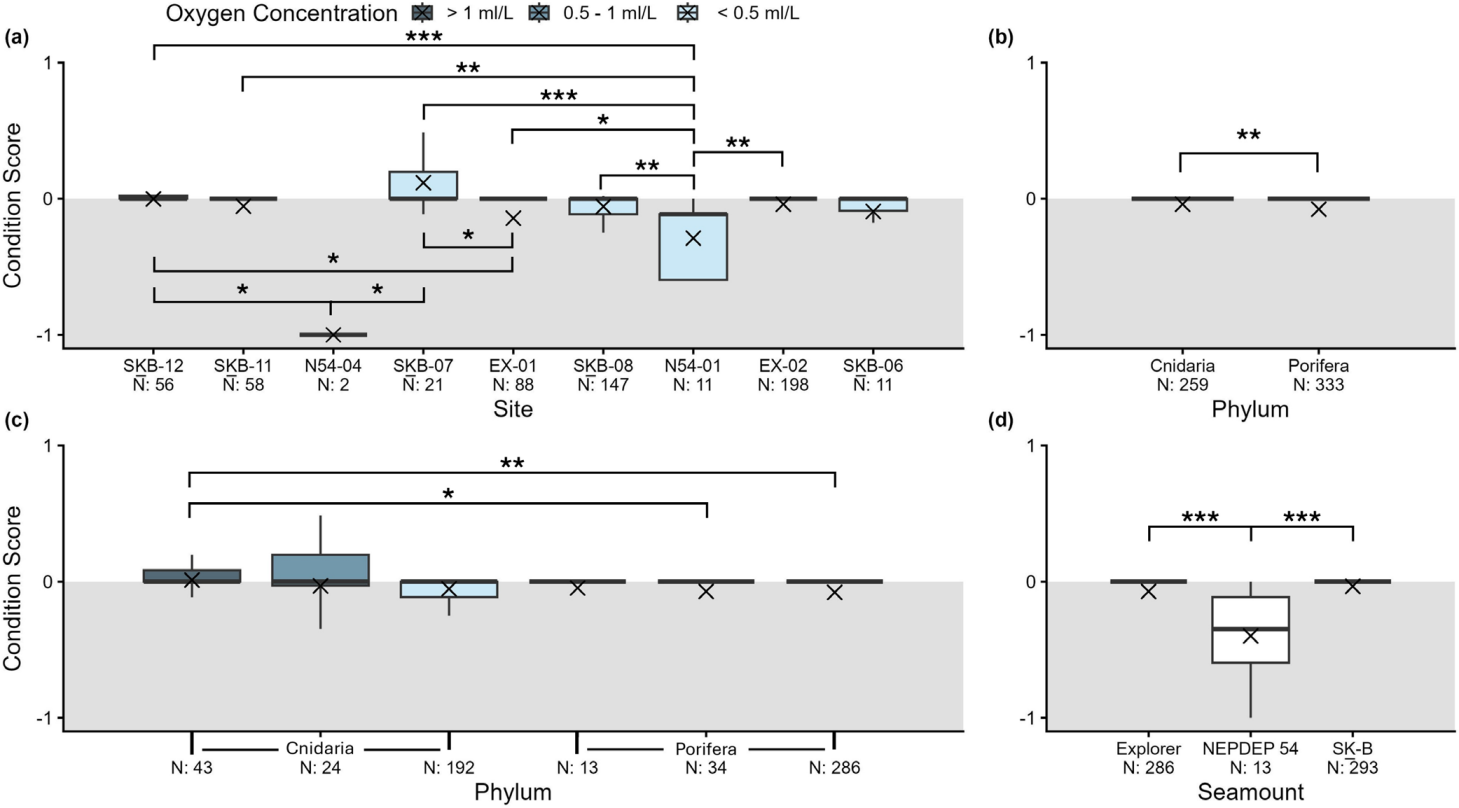
Condition scores at T_1_ observed in long‐term monitoring sites (a) for cold‐water corals (Cnidaria) and sponges (Porifera) (b, c) situated on three Northeast Pacific seamounts: NEPDEP 54, Explorer, and SG̲áan K̲ínghlas‐Bowie (SK̲‐B) (d). Sample sizes (N) represent the number of individuals assessed per factor (i.e., site, phylum, seamount), sites are arranged by descending depth (left to right), and the colour scale represents oxygen concentrations as defined by thresholds for the region's Oxygen Minimum Zone in Ross et al. (2020). Asterisks (*) indicate significant differences as determined by statistical analyses (*: *p* ≤ 0.05, **: *p* ≤ 0.01, ***: *p* ≤ 0.001).

Changes in the condition of corals and sponges occurred at nine of the 12 long‐term monitoring sites examined, with more negative condition changes (21% of observations) observed than positive (13% of observations; Figure 4b, Table S4). On average, 22 of the individuals assessed per site experienced a condition change (positive or negative), with ~36% of these individuals experiencing some level of negative change over time. Mean condition per OTU ranged from −0.15 to 0.049 at T_1_ and mean condition was found to be significantly greater at T_0_ (*p*‐value: 0.0064). Significant differences in the condition of individuals were also detected between sites, phyla, oxygen concentration zones, and seamounts (Figure 7).

Only site SK̲B‐07 had more positive changes than negative (0.12 ± 0.055), while for all other sites, negative changes outweighed any gains (Figure 7a). The most negative condition score (−1) occurred at seven sites (did not occur at SK̲B‐07 and N54‐01), while the most positive condition change (score: 0.75) was observed only twice (SK̲B‐07: 1 individual, SK̲B‐08: 1 individual). Significant differences in condition were detected between long‐term monitoring sites (*p* < 0.001) and the *post hoc* Dunn's test revealed significance between numerous sites. Notably, the most significant differences occurred between N54‐01 and SK̲B‐07 (*p* < 0.001), as well as between N54‐01 and SK̲B‐12 (*p* < 0.001).

Corals (−0.040 ± 0.018) and sponges (−0.077 ± 0.014) largely experienced negative change, but sponges were more sensitive overall (*p*‐value: 0.0036). We further found that sponge condition at T_1_ was worse (i.e., more negative) than corals for each oxygen concentration zone. No significance was found for either intra‐phyla comparisons between the three oxygen concentration zones (Cnidaria: *p* = 0.22, Porifera: *p* = 0.29) or for inter‐phyla comparisons within oxygen concentration zones ([O_2_] > 1 mL/L: *p* = 0.61, 1 mL/L > [O_2_] > 0.5 mL/L: *p* = 0.26, [O_2_] < 0.5 mL/L: *p* = 0.095). Inter‐phyla comparisons between oxygen zones, however, revealed corals located in sites with [O_2_] > 1 mL/L had a significantly greater condition score compared to poriferans in sites within the 1 > [O_2_] > 0.5 mL/L (*p*‐value: 0.0051) and [O_2_] < 0.5 mL/L zones (*p*‐value: 0.00017).

The three OMZ zones hosted animals which declined in condition whereby the mean (± 1 SE) condition was −0.0018 ± 0.031 for sites within the [O_2_] > 1 mL/L zone, −0.055 ± 0.036 for sites classified as 1 > [O_2_] > 0.5 mL/L, and −0.068 ± 0.013 for [O_2_] < 0.5 mL/L sites. We found significance between oxygen concentration zones (Kruskal–Wallis test: *p*‐value: 0.0130) and the *post hoc* Dunn's test determined this significance was driven by differences between sites in the [O_2_] > 1 mL/L and [O_2_] < 0.5 mL/L zones (*p*‐value: 0.0033). We further detected negative condition changes at sites on the three seamounts. Sites on NEPDEP 54 had greater declines than sites on either SK̲‐B or Explorer seamounts (SK̲‐B: −0.035 ± 0.017, Explorer: −0.072 ± 0.014, NEPDEP 54: −0.40 ± 0.099), with the significant differences detected between each seamount pair (NEPDEP 54 vs. Explorer: *p* < 0.001, NEPDEP 54 vs. SK̲‐B: *p* < 0.001).

## 4 Discussion

Evidence of early‐stage range contractions in the marine environment, especially in the deep sea, is exceedingly rare. Here, we detect both population decreases and deteriorating performance in cold‐water corals and sponges on Northeast Pacific seamounts, suggestive of early impacts of environmental change in these deep‐sea ecosystems (although causation has not been established). Over just a span of 3–5 years, five of the 12 long‐term monitoring sites lost 163 individuals between T_0_ and T_1_. At nine sites, the animals that remained declined in condition (e.g., increased tissue loss or biofouling). Sites distinguished as having both abundance and condition losses combined so that two thirds of the population declined overall. A few positive changes in both abundance (+12 individuals across four sites) and condition (13% of individuals present at T_1_) were observed, but these gains were negligible against the broader backdrop of decline.

A key question is thus what is driving these declines? We did not detect consistent trends across sites which fell in different areas of the OMZ, using the instrumentally derived 2018 ROV oxygen data. Our findings do, however, reveal an unequal distribution of condition decline by both phylum and seamount. Corals returned largely positive condition changes, while sponges suffered pronounced declines. Further, the most impacted sites were not always located in the lowest oxygen zone ([O_2_] > 0.5 mL/L) and the seamount closest to the continental margin (NEPDEP 54), where productivity is highest (Du Preez and Norgard 2022), experienced disproportionately greater negative change.

While deoxygenation is recognized as a key threat to cold‐water corals and sponges (Morato et al. 2020; Ross et al. 2020), impacts can be ameliorated, particularly in highly productive regions where energy supplements the cost of physiological mechanisms to overcome stress (Hanz et al. 2019). Given that all three seamounts included within this study are situated within coastal upwelling or transitional zones, characterized by elevated productivity, mixing, and biodiversity (Checkley and Barth 2009; Du Preez and Norgard 2022), how benthic foundational species respond to these key threats may not be straightforward to predict. Seamounts can actively enhance upwelling and stimulate surface primary production (Dower and Fee 1999; Mashayek et al. 2024); indeed, SK̲‐B, with its summit just 24 m below the surface, supports in‐situ primary production (Canessa et al. 2003). This elevated surface productivity and associated organic matter to the seafloor may partially buffer corals and sponges from some of the impacts of deoxygenation, analogous to cold‐water coral reefs and associated sponges off the Angolan coast which thrive in an OMZ (0.6–1.5 mL/L; Hebbeln et al. 2019) sustained by coastal upwelling‐driven productivity (Hanz et al. 2019). The paleorecord similarly points to food availability and turbulent hydrodynamics as the strongest predictors of coral health and longevity over the last 20,000 years (Portilho‐Ramos et al. 2022). Nevertheless, enhanced productivity is unlikely to offset the region's projected deoxygenation trajectory (Franco et al. 2022; Peña and Fine 2024). Further, due to their life history traits (e.g., long lifespans, slow growth rates), the rapid pace of oceanographic condition changes within the region (15% oxygen loss in 60 years; Ross et al. 2020) makes the continued decline of cold‐water corals and sponges on Northeast Pacific seamounts probable. Critically, although sites did not change oxygen concentration zones between T_0_ and T_1_, we did not directly quantify oxygen changes within our sampling intervals due to uncertainty surrounding precise absolute measurements between different instruments. Integrating such in situ oceanographic measurements, alongside particulate organic carbon (POC) flux, with biological observations will be essential for predicting future trajectories.

Sponges have been widely predicted as climate change “winners” relative to corals (Beazley et al. 2021; Bell et al. 2018; Gregório et al. 2024) yet their hypoxia tolerance varies considerably with the degree (severity and length) of oxygen depletion and species identity (Leys and Kahn 2018; Micaroni et al. 2022; Riisgård 2024). Our results further challenge this narrative. Sponges on Northeast Pacific seamounts experienced markedly greater negative change than corals, most strikingly at site EX‐01—named “Spongetopia” during the 2018 expedition. A mass‐mortality event unfolded over the 5 years between photogrammetric surveys (Figure 6): 54% of all sponges were lost from the site, including ~80% of the dominant species *Pinulasma* n. sp. and ~20% of survivors showed negative condition changes. Glass sponges, the predominant deep‐seamount sponge group (including *Pinulasma* n. sp), are rarely found at oxygen concentrations below 1–2 mL/L (Leys et al. 2004), and oxygen concentrations of ~0.1 mL/L, seen at several monitoring sites in 2018, are approaching the limit for sustaining deep‐sea megafauna such as sponges (Gooday et al. 2010; Micaroni et al. 2022; Wishner et al. 1990). Further, while sponges can rapidly recover from short‐term hypoxic events (Micaroni et al. 2022; Riisgård 2024), exposure to deoxygenated conditions result in energetic trade‐offs, including reduced pumping and feeding activity (Leys and Kahn 2018). Prolonged exposure or severe deoxygenation (e.g., lethal thresholds < 0.27 mL/L for two demosponge species; Micaroni et al. 2022) therefore pose an increased risk for performance declines and mortality. As such, declines for this group, particularly within the nearly anoxic core of the OMZ, were expected. The magnitude of loss, however, was not, and thus fundamentally undermines the notion that sponges will thrive through ocean deoxygenation. This result highlights the vulnerability of sponges to changing environmental conditions within the Northeast Pacific, although the specific drivers of this mass mortality event remain to be determined.

Habitat suitability for cold‐water corals is projected to decline globally under continued climate change (Gasbarro et al. 2022; Morato et al. 2020; Rakka et al. 2025). Yet corals on Northeast Pacific seamounts are faring better than expected relative to sponges, particularly at higher oxygen sites (i.e., SK̲‐B‐11 and SK̲‐B‐12), possibly reflecting a lagged response. For instance, following the Deepwater Horizon oil spill in 2010, *Paramuricea* spp. lost branches and continued to decline for 7 years following exposure (Girard and Fisher 2018), and ~40%–50% of large gorgonians on mesophotic reefs had prolonged health declines for several years following the event, with recovery projected to take a decade or more (Etnoyer et al. 2016; Girard et al. 2018). Current monitoring frameworks for deep sea MPAs in Canada similarly recognize that oceanographic impacts on corals and sponges may not manifest for years to decades due to their slow life histories (Du Preez et al. 2024; Kenchington 2010). However, given that we detected substantial changes in just 3–5 years, we recommend frequent surveys to track trajectories effectively and attribute drivers of change. The MPA's monitoring program has already expanded to dozens of additional sites, with some surveyed up to four times since 2018 (Du Preez et al. 2024; Du Preez and Schubert 2025), but continuation of this research is uncertain and subject to national priorities and funding.

Given that NEPDEP 54 has among the highest summit export productivity of any seamount within the Canadian EEZ (48.7 mg C m^−2^ d^−1^; Du Preez and Norgard 2022) and was surveyed over the shortest time interval (i.e., 3 years), the disproportionately low condition scores at sites on this seamount were unexpected. We attribute this largely to methodological limitations of the T_1_ survey of these sites, which used the drop camera BOOTS in an opportunistic “fly‐by” approach, yielding only 7%–43% site coverage versus 75%–100% coverage at sites on the other seamounts. Poor lighting, vignetting, and limited vehicle control needed to position the camera during surveys resulted in variable viewing angles compared to the 2018 imagery. Thus capacity to observe small, cryptic individuals within the site and detect subtle condition changes likely reduced sample sizes and inflated apparent negative scores (Type I error risk; Faber and Fonseca 2014; Serdar et al. 2021). Further, measurements used for traditional condition metrics, such as branch number and length, surface area, or volume (e.g., Bennecke et al. 2016; Girard and Fisher 2018; Olinger et al. 2019), were unable to be obtained via the two‐dimensional mosaics due to the changes in camera orientation and organism visibility between years (also present in ROV imagery, but to a lesser extent) resulting in the qualitative assessment used within this study. Repeat surveys for NEPDEP 54 sites, preferably using ROVs or similar equipment to minimize differences in positioning, lighting, and viewing angle, as well as efforts to quantitatively assess condition changes within all monitoring sites, are required to confirm emergent trends.

## 5 Conclusion

Over just three to 5 years, we document significant declines in coral and sponge communities on Northeast Pacific seamounts suggesting these deep‐sea ecosystems may be experiencing rapid early‐stage population declines that, if sustained, contribute to range contractions. However, future work integrating environmental measurements with biological observations will be needed to establish the link between these changes and climate‐driven range contractions. Sponges, long predicted as climate “winners” (Beazley et al. 2021; Gregório et al. 2024), fared significantly worse than cold‐water corals, while results suggest potential lag effects in coral disturbance responses (Etnoyer et al. 2016; Girard and Fisher 2018). Thus, the full extent of ocean change impacts on these species may not yet be apparent. The lack of consistent trends with oxygen concentration underscores that additional stressors, or site‐level buffering by productivity, may be moderating outcomes. Establishing causation will require integrating in situ measurements of oceanographic conditions, including oxygen and particulate organic carbon flux, paired with biological time‐series to better understand the primary drivers of change. Repeat monitoring efforts are essential for resolving the dynamics of change in these long‐lived, slow‐growing species. As ocean change accelerates species local extinction events globally (Gilson et al. 2021; Pinsky and Fredston 2022), sustained monitoring of deep‐sea foundation species is not merely a scientific imperative. Instead, we advocate that sustained monitoring is a prerequisite for vulnerable deep‐sea habitats and will be essential for informing effective marine protected area (MPA) management and ocean policy.

## Author Contributions

**Lindsay Clark:** conceptualization, data curation, formal analysis, investigation, methodology, validation, visualization, writing – original draft, writing – review and editing. **Georgia Clyde:** writing – review and editing, visualization, validation, resources, methodology, investigation, formal analysis, data curation. **Cherisse Du Preez:** writing – review and editing, visualization, validation, supervision, resources, methodology, investigation, funding acquisition, conceptualization. **Amanda E. Bates:** writing – review and editing, writing – original draft, visualization, validation, supervision, methodology, funding acquisition, conceptualization.

## Funding

Research was funded by Fisheries and Oceans Cananda (Deep‐sea Ecology Program).

## Conflicts of Interest

The authors declare no conflicts of interest.

## Supporting information


**Table S1:** Frequency‐weighted and severity‐scaled frequency‐weighted scores for condition metrics, scaled between zero and one, used to determine overall change in condition between T_0_ (2018) and T_1_ (site specific) for corals and sponges in 12 long‐term monitoring sites on Northeast Pacific seamounts.
**Table S2:** Output parameters of 24 photogrammetric reconstructions of long‐term monitoring sites on three Northeast Pacific seamounts, NEPDEP 54 (N54), Explorer (EX), and SG̲áan K̲ínghlas‐Bowie (SK̲‐B).
**Table S3:** Comparison of T_0_ (2018) and T_1_ (site dependent) mosaics derived from reconstructions of long‐term monitoring sites on three Northeast Pacific seamounts, NEPDEP 54 (N54), Explorer (EX), and SG̲áan K̲ínghlas‐Bowie (SK̲‐B).
**Table S4:** Abundance changes and percent of individuals with condition changes for cold‐water corals and sponges between T_0_ and T_1_ for 12 long‐term monitoring sites on NEPDEP 54 (N54), Explorer (EX), and SG̲áan K̲ínghlas‐Bowie (SK̲‐B) seamounts.

## Data Availability

All raw video imagery data is stored and available through Ocean Networks Canada's SeaTube Pro: https://data.oceannetworks.ca/SeaTube. All open‐source software used during the analyses are available online: GIMP (https://www.gimp.org/), BIMP (https://alessandrofrancesconi.it/projects/bimp/), R (https://www.R‐project.org/), RStudio (http://www.posit.co/), QGIS (https://qgis.org/en/site/), ImageCaptureTool (https://gitlab.com/dfo‐msea/rov‐annotation/‐/tree/master/image‐annotation?ref_type=heads), ExifTool (https://exiftool.org/), and Geosetter (https://geosetter.de/en/main‐en/). R code and workflows used for the creation of reconstructions and the statistical analyses described herein are openly available on GitHub (https://github.com/Lclark17/Clark_etal_2026_Rapid‐decreases) and Figshare (https://doi.org/10.6084/m9.figshare.30604394).
