# Supplementary material for: Rapid Decreases and Performance Declines in Northeast Pacific Seamount Foundation Species Detected in an Oxygen Minimum Zone
Source: Glob Chang Biol. 2026 Apr 27;32:e70878. doi: 10.1111/gcb.70878 (PMC13112330; doi:10.1111/gcb.70878)
Supplement: Supplementary file 1 — Table S1: Frequency‐weighted and severity‐scaled frequency‐weighted scores for condition metrics, scaled between zero and one, used to determine overall change in condition between T0 (2018) and T1 (site specific) for corals and sponges in 12 long‐term monitoring sites on Northeast Pacific seamounts. Table S2: Output parameters of 24 photogrammetric reconstructions of long‐term monitoring sites on three Northeast Pacific seamounts, NEPDEP 54 (N54), Explorer (EX), and SG̲áan K̲ínghlas‐Bowie (SK̲‐B). Table S3: Comparison of T0 (2018) and T1 (site dependent) mosaics derived from reconstructions of long‐term monitoring sites on three Northeast Pacific seamounts, NEPDEP 54 (N54), Explorer (EX), and SG̲áan K̲ínghlas‐Bowie (SK̲‐B). Table S4: Abundance changes and percent of individuals with condition changes for cold‐water corals and sponges between T0 and T1 for 12 long‐term monitoring sites on NEPDEP 54 (N54), Explorer (EX), and SG̲áan K̲ínghlas‐Bowie (SK̲‐B) seamounts. [file GCB-32-e70878-s001.pdf]

Supporting Information accompanying:

Rapid decreases and performance declines in Northeast Pacific seamount foundation species detected in an oxygen minimum zone

Authors: Lindsay Clark[1,2]*, Cherisse Du Preez[1,2], Georgia Clyde[2], and Amanda E. Bates[1]

[1] *Department of Biology, University of Victoria, Victoria, BC, Canada*

[2] *Fisheries and Oceans Canada, Institute of Ocean Sciences, Sidney, BC, Canada*

* *Corresponding author*

Table S1. Frequency-weighted and severity-scaled frequency-weighted scores for condition metrics, scaled between zero and one, used to determine overall change in condition between T_0 (2018) and T_1 (site specific) for corals and sponges in 12 long-term monitoring sites on Northeast Pacific seamounts.

**Initial Score**	**Frequency-Weighted Score**			
	**Growth**	**Tissue Loss**	**Biofouling**	**Vertical Orientation Change**
0	0	0	0	0
1	0.7894737	0.4553571	1	0.3529412
2	0.9736842	0.6964286	-	1
3	1	0.7946429	-	-
4	-	1	-	-

**Initial Score**	**Severity-Scaled Frequency-Weighted Score**			
	**Growth**	**Tissue Loss**	**Biofouling**	**Vertical Orientation Change**
0	0	0	0	0
1	0.1973684	0.1138393	0.25	0.1764706
2	0.4868421	0.3482143	-	1
3	0.75	0.5959821	-	-
4	-	1	-	-

Table S2. Output parameters of 24 photogrammetric reconstructions of long-term monitoring sites on three Northeast Pacific seamounts, NEPDEP 54 (N54), Explorer (EX), and SG̱áan Ḵínghlas-Bowie (SḴB).

Site	Year	Resolution (cm/pixel)	Camera Optimization (%)	Computed Error Length (m)	Absolute Geolocation RMSE (m)
N54-01	2018	0.30	6.7	0.044	-
	2021	0.19	2.8	-	**X:** 0.28, **Y:** 1.4, **Z:** 2.0
N54-02	2018	0.36	5.1	0.061	-
	2021	0.43	0.84	-	**X:** 0.19, **Y:** 0.17, **Z:** 0.31
N54-03	2018	0.38	6.3	0.0018	-
	2021	0.45	1.6	-	**X:** 0.26, **Y:** 0.13, **Z:** 0.086
N54-04	2018	0.35	8.7	0.029	-
	2021	0.53	2.0	-	**X:** 0.85, **Y:** 0.50, **Z:** 0.30
N54-06	2018	0.27	3.8	0.041	-
	2021	1.0	0.61	-	**X:** 0.20, **Y:** 0.36, **Z:** 0.15
SḴB-06	2018	0.42	1.7	0.083	**X:** 4.2, **Y:** 1.8, **Z:** 3.6
	2022	0.30	10	0.23	**X:** 1.9, **Y:** 1.6, **Z:** 0.62

Site	Year	Resolution (cm/pixel)	Camera Optimization (%)	Computed Error Length (m)	Absolute Geolocation RMSE (m)
SḴB-07	2018	0.30	2.7	0.0026	**X:** 2.1, **Y:** 1.7, **Z:** 3.3
	2022	0.17	1.4	0.0020	**X:** 0.52, **Y:** 0.48, **Z:** 0.43
SḴB-08	2018	0.37	4.1	0.096	**X:** 2.4, **Y:** 2.3, **Z:** 3.6
	2022	0.36	0.56	0.17	**X:** 1.8, **Y:** 1.7, **Z:** 2.1
SḴB-11	2018	0.24	5.8	-0.038	**X:** 1.0, **Y:** 0.78, **Z:** 0.25
	2022	0.31	1.7	0.14	**X:** 1.3, **Y:** 1.1, **Z:** 0.53
SḴB-12	2018	0.42	1.9	0.069	**X:** 0.87, **Y:** 0.46, **Z:** 0.47
	2022	0.43	12	-0.062	**X:** 1.4, **Y:** 0.77, **Z:** 0.56
EX-01	2018	0.52	5.2	-0.025	**X:** 2.7, **Y:** 5.2, **Z:** 2.7
	2023	0.72	13	-0.073	**X:** 1.8, **Y:** 3.0, **Z:** 0.65
EX-02	2018	0.51	0.13	0.035	**X:** 1.2, **Y:** 0.82, **Z:** 0.11
	2023	0.43	4.4	-0.037	**X:** 0.93, **Y:** 0.75, **Z:** 0.086

Table S3. Comparison of T_0 (2018) and T_1 (site dependent) mosaics derived from reconstructions of long-term monitoring sites on three Northeast Pacific seamounts, NEPDEP 54 (N54), Explorer (EX), and SG̱áan Ḵínghlas-Bowie (SḴB).

Site	T_0 Reconstruction Area (m^2)	Overlap Area (m^2)	Portion of Site Re-surveyed (%)	Georeferencing RMS Error (cm)
N54-01	119	8.13	6.84	3.39
N54-02	184	32.7	17.8	6.40
N54-03	154	26.6	17.3	6.08
N54-04	164	70.7	43.2	17.3
N54-06	136	29.9	21.9	7.93
SḴB-06	220	220	100	30.4
SḴB-07	253	236	93.1	10.3
SḴB-08	204	186	91.2	72.6
SḴB-11	181	180	99.7	82.6
SḴB-12	366	275	75.0	74.2
EX-01	183	183	100	97.3
EX-02	345	345	100	25.2

Table S4. Abundance changes and percent of individuals with condition changes for cold-water corals and sponges between T_0 and T_1 for 12 long-term monitoring sites on NEPDEP 54 (N54), Explorer (EX), and SG̲áan K̲ínghlas-Bowie (SK̲B) seamounts.

Site	T_0 Abundance	Abundance Change at T_1	Individuals Assessed for Condition Change	Negative Condition Changes (%)	Positive Condition Changes (%)
N54-01	39	0	11	81.8	-
N54-02	5	0	-	-	-
N54-03	4	0	-	-	-
N54-04	17	0	2	100	-
N54-06	6	0	-	-	-
SK̲B-06	11	0	11	27.3	18.2
SK̲B-07	21	0	21	14.3	38.1
SK̲B-08	163	-15 (-16/+1)	147	31.3	22.4
SK̲B-11	73	-9 (-15/+6)	58	22.4	13.8
SK̲B-12	76	-13	56	10.7	25.0
EX-01	190	-97 (-98/+1)	88	19.3	-
EX-02	239	-29 (-33/+4)	198	13.6	5.1
